# Dual-Setting Magnesium Phosphate Bone Cement with Improved Handling Through the Integration of Chitosan-Derived Hydrogels

**DOI:** 10.3390/jfb17100481

**Published:** 2026-09-22

**Authors:** Marcin Wekwejt, Kaja Stanisławska, Dawid Kozień, Anna Ronowska, Justyna Kozłowska

**Affiliations:** 1 Biomaterials Technology Department, Faculty of Mechanical Engineering and Ship Technology, Gdańsk University of Technology, 80-233 Gdańsk, Poland; 2 Scientific Club ‘Materials in Medicine’, Advanced Materials Centre, Gdańsk University of Technology, 80-210 Gdańsk, Poland; s196251@student.pg.edu.pl; 3 Faculty of Materials Science and Ceramics, AGH University of Krakow, 30-059 Krakow, Poland; kozien@agh.edu.pl; 4 Department of Laboratory Medicine, Medical University of Gdańsk, 30-059 Gdańsk, Poland; anna.ronowska@gumed.edu.pl; 5 Faculty of Chemistry, Nicolaus Copernicus University in Toruń, 87-100 Toruń, Poland; justynak@umk.pl; 6 Institute of Advanced Studies, Nicolaus Copernicus University in Toruń, 87-100 Toruń, Poland

**Keywords:** magnesium phosphate cement, dual-setting biomaterial, chitosan hydrogel, β-glycerophosphate, setting kinetics, minimally invasive surgery, bone regeneration

## Abstract

Magnesium phosphate cements (MPCs) are promising bone substitutes, but rapid setting, high exothermicity, and poor handling limit their clinical application. This study developed a dual-setting MPC by integrating chitosan-derived hydrogels into the cement matrix. Zinc ion-crosslinked carboxymethyl chitosan (CMCS) and thermosensitive chitosan/β-glycerophosphate (CS/GP) hydrogels were initially evaluated as modifiers of borax-containing (0.5% B) MPC formulations, after which the CS/GP formulation was further optimized by varying the CS (1.0/1.5%) and GP (5.6%/8.0%) concentrations. The effects of these modifications on setting behavior, physicochemical characteristics, mechanical performance, biodegradation, and cytocompatibility were systematically investigated. The B/CS/GP systems prolonged setting time, reduced the reaction temperature, moderated pH evolution, and markedly improved qualitative injectability and cohesion. XRD, FTIR, and SEM confirmed preservation of the characteristic phase composition, chemical structure, and microstructure. All formulations maintained favorable mechanical properties and supported osteoblast viability above the threshold. Among the investigated groups, the cement containing 0.5% B, 1.5% CS, and 5.6% GP exhibited the most balanced performance, combining improved handling characteristics with enhanced compressive strength. These findings demonstrate that the integration of CS-derived hydrogels, particularly the thermosensitive CS/GP system, provides a promising strategy for developing injectable dual-setting MPCs with improved handling characteristics without compromising their fundamental physicochemical and biological properties.

## 1. Introduction

Bone possesses a remarkable capacity for self-repair; however, this regenerative potential becomes insufficient in critical-sized defects caused by trauma and tumor resection as well as in infection or metabolic bone diseases. In such situations, surgical intervention and biomaterial-assisted regeneration are often required to restore both structural integrity and biological function [1,2,3]. Injectable self-setting bone substitutes have therefore attracted considerable attention because they enable minimally invasive implantation, conform to irregular defect geometries, and establish intimate contact with the surrounding tissue while avoiding the drawbacks associated with pre-formed implants [4,5].

Among injectable biomaterials, self-setting bone cements represent one of the most clinically relevant classes owing to their ability to be delivered as moldable pastes that harden directly within the defect site [6,7]. Their clinical performance depends not only on the properties of the hardened material but also on handling characteristics before implantation [4]. An ideal injectable cement should exhibit adequate setting time, injectability, and cohesion, harden under physiological conditions without excessive heat release or unfavorable pH fluctuations, and gradually degrade in concert with new bone formation [4,5,7]. Simultaneously satisfying these often competing requirements remains a major challenge in the development of next-generation bone cements.

Magnesium phosphate cements (MPCs) have emerged as a promising alternative to conventional bone cements (calcium phosphate (CP)- and poly(methyl methacrylate) (PMMA)-based) because of their rapid strength development, biodegradability, and ability to release biologically beneficial Mg^2+^ ions that promote osteogenesis and bone remodeling [8,9,10]. These advantages have stimulated increasing interest in MPC, particularly for the treatment of osteoporotic and non-load-bearing bone defects [8,11]. Nevertheless, the rapid acid-base reaction responsible for their early hardening also restricts working time and may result in undesirable heat generation and transient pH changes [8,12]. Moreover, injectable MPC formulations must maintain sufficient cohesion and resistance to washout before complete setting without compromising the characteristic cement chemistry [9,11].

To overcome these limitations, numerous modification strategies have been explored, including setting retarders, optimization of the Mg-to-P molar ratio or the powder-to-liquid ratio, bioactive additives, and polymeric modifiers [8,11,13]. Among these approaches, borax is widely used to prolong working time while preserving characteristic reaction products [14]. Furthermore, hydrogel-assisted systems have attracted growing interest because they improve paste rheology, cohesion, and extrusion behavior while generally preserving the inorganic hydration products of MPC [15,16,17]. However, in nearly all reported systems, polymers primarily function as rheological modifiers or viscosity-enhancing additives. Their contribution has therefore been attributed mainly to particle coating, improved matrix continuity, or physical interactions with cement constituents [18,19], rather than to the formation of an independently evolving polymer network.

Chitosan (CS) is one of the most extensively investigated natural polymers for biomedical applications owing to its excellent biocompatibility, biodegradability, and hydrogel-forming capability [20,21,22]. Consequently, native CS and its derivatives have been investigated as modifiers for MPC formulations. Previous studies demonstrated that incorporating CS, carboxymethyl CS (CMCS), or O-CMCS can prolong setting time, reduce the exothermic reaction, improve cohesion, and enhance mechanical performance while generally preserving the characteristic phase composition and hydration products of the MPC [19,23,24,25]. Blended polysaccharide systems, including CMCS/sodium alginate, have further improved handling characteristics and biological performance [26]. Despite these advances, existing studies have predominantly focused on polymer-modified MPC rather than on systems in which polymer gelation actively participates in the setting process.

An alternative concept is provided by dual-setting systems, in which hydrogel gelation and cement hydration occur simultaneously and cooperatively. Such an approach has the potential to integrate the advantages of both inorganic cement hardening and hydrogel network formation, thereby providing superior handling properties while preserving the biological and mechanical characteristics of MPC. In this context, CS offers an additional advantage over conventional polymers, as its pH-, temperature-, and/or ion-dependent behavior enables it to actively participate in hydrogel network formation rather than acting solely as a passive modifier of the cement matrix. Among hydrogel-forming systems, thermosensitive CS/β-glycerophosphate (β-GP) formulations are particularly attractive because they remain injectable at low temperature and undergo sol-to-gel transition under physiological conditions without requiring external cross-linking agents [27,28,29]. In contrast, CMCS-hydrogel relies primarily on ion-mediated cross-linking through dynamic coordination with multivalent metal ions such as Zn^2+^ [30,31,32]. Owing to these fundamentally different gelation mechanisms, both systems are expected to interact differently with the hydration reaction of freshly setting MPC, yet such interactions remain poorly understood.

To address this knowledge gap, the present study developed a dual-setting MPC cement based on CS-derived hydrogels with improved handling and extrusion behavior. Two fundamentally different hydrogel-forming strategies, namely thermosensitive CS/GP and ionically cross-linked CMCS systems, were first comparatively evaluated to identify the most suitable approach for dual-setting MPC design. The selected formulation was subsequently optimized by varying the contents of CS and the cross-linking agent, and was comprehensively characterized with respect to handling properties, setting behavior, physicochemical characteristics, phase composition, microstructure, degradation, mechanical performance, and in vitro cytocompatibility. By directly linking the CS-based hydrogel formulation with cement hydration behavior, this work provides mechanistic insight into the rational design of next-generation injectable dual-setting MPC biocomposites.

## 2. Materials and Methods

### 2.1. Preparation of Dual-Setting Magnesium Phosphate-Chitosan Cements

Dual-setting cement formulations were obtained by combining a reactive magnesium phosphate system with chitosan-based polymeric phases. The powder component consisted of dead-burned magnesium oxide (MgO; average particle size: 9.04 ± 0.44 μm; Thermo Fisher Scientific, Waltham, MA, USA; 1500 °C/6 h under argon atmosphere) and potassium dihydrogen phosphate (KH_2_PO_4_; average particle size: 78.10 ± 0.44 μm; Chempur, Piekary Śląskie, Poland) mixed at a Mg/P molar ratio of 4:1. Sodium tetraborate (borax; 0.5 wt.%; Chempur, Piekary Śląskie, Poland) was incorporated as a setting retarder [33]. Depending on the formulation, β-glycerophosphate (GP) or zinc carbonate (ZnCO_3_) were incorporated to facilitate the formation of the corresponding hydrogel network.

Two chitosan derivatives were investigated as polymeric components: chitosan (CS) and carboxymethyl chitosan (CMCS). The CS solution was prepared by dissolving chitosan in 1% (*v*/*v*) acetic acid (Chempur, Piekary Śląskie, Poland) under continuous magnetic stirring overnight to ensure complete dissolution, whereas CMCS was directly dissolved in distilled water due to its water-soluble nature. Owing to their distinct physicochemical characteristics, different gelation mechanisms were employed. CS-containing formulations underwent β-glycerophosphate (Loba Chemie, Mumbai, India)-mediated thermosensitive gelation (based on [28,34,35]), whereas CMCS-containing formulations relied on zinc-mediated ionic cross-linking (Chempur, Piekary Śląskie, Poland), promoted by the gradual acidification induced by hydraulic reaction, with glucono-δ-lactone (GDL; Thermo Fisher Scientific, Waltham, MA, USA) serving as an additional modulating agent (based on [36]). Consequently, the developed systems combined magnesium phosphate hydration with either thermally triggered or ionically induced polymer network formation, resulting in dual-setting ceramic-hydrogel composites.

The cement paste was prepared by combining both phases at a powder-to-liquid ratio of 2.5 g/mL and manually mixing until a homogeneous injectable paste was formed. Following mixing, the material underwent simultaneous cement hardening and hydrogel formation. The resulting pastes were cast into silicone molds of the desired geometry and cured for 24 h at 36.6 °C under ~90% relative humidity (Figure 1). Detailed compositions of all investigated formulations, including polymer concentration and cross-linking systems, are presented in Table 1.

### 2.2. Setting Behavior

The setting behavior of the developed cements was evaluated by determining setting time, reaction temperature, and pH evolution during hydration. Setting time was measured using a Vicat apparatus (MMC-0450/E, Multiserw-Morek, Marcyporęba, Poland) equipped with a metallic needle (1.13 mm diameter, 300 g load). The final setting time was defined as the period from initial mixing to the disappearance of any visible indentation on the specimen surface. Temperature evolution during cement hardening was monitored using a thermocouple (Cz-Term, Czah, Katowice, Poland). Briefly, cement paste was transferred into a 2 mL Eppendorf tube immediately after mixing, and the maximum temperature reached during the setting reaction was recorded under ambient laboratory conditions. Changes in pH during hydration were assessed semi-quantitatively using universal pH indicator strips (Macherey-Nagel, Dueren, Germany; accuracy ± 0.5 pH units). Measurements were performed every 2 min throughout the setting process. All measurements of setting behavior were performed in triplicate (*n* = 3).

### 2.3. Physicochemical Characterization

The morphology, phase and chemical composition of cured cements were investigated using complementary microscopic and spectroscopic techniques. Microstructural observations were carried out using a high-resolution scanning electron microscope (Apreo 2S, Thermo Fisher Scientific, Waltham, MA, USA). Specimens were manually fractured to expose cross-sections, sputter-coated with a thin gold layer, and examined at magnifications of 500× and 2000×. Chemical characterization was performed using attenuated total reflection Fourier-transform infrared spectroscopy (ATR-FTIR; Nicolet iS5, Thermo Fisher Scientific, Waltham, MA, USA) equipped with a ZnSe crystal. Spectra were acquired over the 4000–500 cm^−1^ range using 64 scans and a spectral resolution of 4 cm^−1^. All spectra were normalized and smoothed prior to interpretation. Phase composition was evaluated by X-ray diffraction (XRD) using a diffractometer equipped with Cu-Kα radiation (PANalytical X’Pert PRO, Philips, Amsterdam, The Netherlands). Prior to analysis, specimens were crushed and ground into powder. Data were collected over a 2θ range of 5–90° using a step size of 0.02°, an operating voltage of 40 kV, and a current of 40 mA. Phase identification was performed using HighScore Plus software (version 4, Malvern Panalytical, Malvern, UK) and the Inorganic Crystal Structure Database (ICSD), with reference patterns corresponding to k-struvite and magnesium oxide.

The initial open porosity of the cured specimens (*n* = 4) was determined gravimetrically according to Equation (1) [36]. Prior to analysis, specimens were dried at 50 °C for 24 h, cooled to room temperature, and subsequently saturated with water. Porosity (Φ) was calculated as follows:

Φ = ((mw − md)/(ρV)) × 100%,(1)

where mw is the saturated mass of the specimen (g), md is the dry mass (g), ρ is the density of the immersion medium (g/cm^3^), and V is the specimen volume (cm^3^).

### 2.4. Mechanical Properties

Mechanical performance was evaluated under uniaxial compression using a universal testing machine (Z005, Zwick & Roell, Ulm, Germany) equipped with a 5 kN load cell. Measurements (*n* = 7–10) were performed at a crosshead speed of 1 mm/min. Compressive strength (σc) and compressive modulus (Ec) were calculated from the resulting stress–strain curves using TestXpert III software.

### 2.5. In Vitro Degradation

Prior to degradation testing, cured specimens (*n* = 4) were rinsed in distilled water for 3 h to remove residual soluble salts from the pore structure. Then, they were dried at 50 °C for 24 h and weighed to determine their initial mass (mi). The specimens were immersed in phosphate-buffered saline (PBS; pH 7.4; Merck, Darmstadt, Germany) and incubated at 37 °C for 28 days. The immersion medium was replaced every 2–3 days. Following incubation, specimens were removed, dried for 24 h, and weighed again to determine the final mass (mf). Relative mass change (m) was calculated according to Equation (2) [36]:

m (%) = (mf/mi) × 100%,(2)

where mi and mf represent the initial and final specimen masses (g), respectively.

### 2.6. Qualitative Assessment of Extrusion Behavior and Cohesion

Injectability and cohesion of the developed formulations were assessed qualitatively under simulated physiological conditions (*n* = 3). Freshly prepared cement pastes were loaded into 5 mL syringes and allowed to rest for approx. 5 min prior to extrusion. The materials were subsequently injected into PBS solution, and their ability to maintain structural integrity and resist disintegration was visually evaluated. Representative photographs were recorded 15 min after extrusion.

### 2.7. Cytocompatibility

The biological performance of the developed cements was evaluated using the human fetal osteoblast cell line hFOB 1.19 (RRID: CVCL_3708; ATCC, Manassas, VA, USA). Cells were cultured in DMEM/F-12 medium (Gibco, Grand Island, NY, USA) supplemented with 10% fetal bovine serum (Biowest, Nuaillé, France) and 0.3 mg/mL geneticin sulfate (ThermoFisher Scientific, Waltham, MA, USA) under standard culture conditions: 34 °C, 5% CO_2_, 95% humidity. Prior to biological evaluation, all specimens were sterilized by UV irradiation (2 × 30 min) and preconditioned in culture medium for 7 days to reduce ion depletion from the medium and establish equilibrium at the material-medium interface [37,38]. For cytocompatibility assessment, hFOB cells were seeded directly onto specimen surfaces (*n* = 4) at a density of 80 × 10^3^ cells/mL in 1.5 mL of fresh culture medium. After 3 days of incubation, cell metabolic activity was quantified using the MTT (thiazolyl blue tetrazolium bromide) assay (Merck, Darmstadt, Germany). Absorbance was measured at 595 nm with a reference wavelength of 690 nm using a microplate reader (Victor, PerkinElmer, Shelton, CT, USA). Results were normalized to cells cultured on tissue culture polystyrene (TCP), which served as a reference control.

### 2.8. Statistical Analysis

Statistical analysis was performed using SigmaPlot software, version 15.0 (Systat, San Jose, CA, USA). Data normality was verified using the Shapiro–Wilk test. Results are presented as mean ± standard deviation (SD). Statistical significance was assessed using one-way analysis of variance (ANOVA) followed by Bonferroni’s post hoc test. Differences were considered statistically significant at *p* < 0.05.

## 3. Results

The developed magnesium phosphate cements, modified with CS and CMCS, were first screened for setting behavior, paste cohesion, and qualitative injectability. Based on the obtained results, only the CS-containing formulations demonstrated sufficient performance to warrant further investigation and were therefore subjected to comprehensive physicochemical, mechanical, degradation, and biological characterization.

### 3.1. Influence of Chitosan Derivatives on the Early Performance of MPC

#### 3.1.1. Setting Time

The setting time results are summarized in Table 2A,B. Borax incorporation extended the setting time by approx. 3.5 min compared with the reference MPC. Further modifications with CS and CMCS resulted in setting times ranging from approx. 21 to 26 min. While most formulations exhibited setting times comparable to those of the borax-modified cements, MPC_B+1.0%CS+8.0%GP and MPC_B+1.5%CMCS+200Zn showed a further significant prolongation of the setting time. No clear concentration-dependent trend was observed within either the CS or CMCS series.

#### 3.1.2. Reaction Temperature

The maximum reaction temperature values are presented in Table 2A,B. Borax incorporation reduced the reaction temperature by approx. 14 °C compared with the reference MPC. The CS-containing formulations maintained temperatures comparable to those of the borax-modified cement, ranging from approx. 38 to 40 °C. In contrast, all CMCS-modified formulations exhibited significantly higher reaction temperatures than MPC_B, reaching values between approx. 49 and 52 °C. Within each tested series, the reaction temperature remained relatively consistent across all formulations.

#### 3.1.3. Extrusion Behavior and Cohesion

Representative images of extruded strands are presented in Figure 2. All formulations could be successfully extruded from the syringe; however, clear differences in post-extrusion behavior were observed. Compared with the reference MPC, borax incorporation resulted in increased strand spreading and reduced shape retention. The addition of CS improved post-extrusion strand integrity and cohesion, resulting in more coherent, better-defined structures. In contrast, CMCS-modified formulations exhibited reduced cohesion accompanied by increased strand deformation and poorer shape retention.

#### 3.1.4. Selection of Formulations for Subsequent Studies

Based on the results obtained during the initial screening, the CS-containing formulations were selected for further investigation. Compared with CMCS-modified cements, these formulations exhibited lower reaction temperatures and improved post-extrusion cohesion under wet conditions, while maintaining comparable setting times.

Since no substantial differences in early performance were observed among the CS-containing formulations, none of the investigated CS or GP concentrations were excluded at this stage. Therefore, subsequent physicochemical, mechanical, degradation, and biological evaluations were performed exclusively on the CS-containing cements.

### 3.2. Physicochemical Characterization

#### 3.2.1. Microstructure Analysis

Representative SEM micrographs of the developed cements are presented in Figure 3. The reference MPC exhibited a heterogeneous microstructure, predominantly composed of densely packed granular agglomerates and hydration product crystals embedded within a dense gel-like matrix. The incorporation of borax did not cause noticeable alterations in the overall microstructure compared with the control. Similarly, the addition of CS hydrogel maintained the integrity of the cement matrix, with no visible phase separation between the ceramic and polymer components. Most formulations exhibited fine granular features; however, localized regions containing larger plate-like and rosette-like crystalline structures were observed, particularly in cements containing 8.0%GP. No pronounced macropores or large voids were observed in the analyzed specimens, and all formulations exhibited a relatively dense microstructure at the examined magnifications. Moreover, microcracks were found in all formulations—these defects are likely related to specimen preparation (cross-section fracture) and may have been further accentuated by drying prior to SEM examination.

#### 3.2.2. pH Evolution of Cement Pastes

All tested formulations exhibited a rapid increase in pH during the first minute after mixing, indicating progressive neutralization of the initially acidic phosphate solution (Figure 4). The highest initial pH value was observed for MPC+1.5%CS, whereas borax-containing formulations showed a slower increase in pH. The incorporation of GP further moderated the initial alkalization rate; however, no noticeable differences were observed between the tested GP concentrations. Despite these variations during the early stages of setting, all formulations exhibited a similar pH evolution profile and reached a comparable final pH value (~7.5) within 10–15 min.

#### 3.2.3. Phase and Chemical Characterization

The XRD patterns of the tested cements are presented in Figure 5A, while quantitative phase composition is summarized in Table 3. All formulations exhibited similar diffraction profiles and were composed predominantly of k-struvite and residual MgO. No additional crystalline phases were detected following the incorporation of B, CS, or GP. Quantitative analysis revealed only minor variations in phase composition among the investigated groups, with k-struvite content ranging from 60.6 to 61.2% and MgO content from 38.8 to 39.4%.

The FTIR spectra of the tested cements are presented in Figure 5B. All formulations exhibited similar absorption profiles, indicating that the incorporation of CS and GP did not substantially alter the chemical structure of the hardened cement matrix. Characteristic bands associated with O-H stretching (~2900 cm^−1^), H-O-H bending (~1600 cm^−1^), phosphate vibrations (990 cm^−1^), O-P-O/P-O-P vibrations (~600 cm^−1^), and Mg-O vibrations (~440 cm^−1^) were observed in all cement groups. Following the incorporation of CS hydrogel, a slight broadening of the high-wavenumber O-H/N-H stretching region was observed, consistent with the overlap of hydroxyl vibrations from the cement matrix and hydroxyl/amino groups of CS. Meanwhile, the characteristic phosphate-related bands remained unchanged. No additional bands corresponding to newly formed chemical species were observed.

Overall, both XRD and FTIR results demonstrated that the incorporation of CS hydrogel preserved the characteristic phase composition and chemical structure of the hardened MPC.

#### 3.2.4. Porosity

The open porosity results are presented in Figure 6. The porosity of the reference formulations (MPC and MPC_B) remained at a comparable level, reaching approx. 5.5%, indicating that the incorporation of borax alone did not significantly affect the pore structure of the hardened cement matrix. The addition of CS hydrogel generally increased the porosity of the cements, suggesting that the formation of a polymer-containing network promoted the development of additional voids within the matrix during the setting process. The highest porosity value was recorded for MPC_B+1.5%CS+8.0%GP. Overall, increasing the CS content resulted in a tendency toward higher porosity, although all tested formulations remained below 8%, reflecting the relatively compact microstructure of the developed cements.

### 3.3. Degradation

The degradation behavior of the tested cements after 28 days of incubation in PBS is shown in Figure 7. All formulations exhibited relatively low mass loss, ranging from approx. 6–8%, indicating good resistance to dissolution under physiological-like conditions. The introduction of CS at a higher concentration resulted in an increase in mass loss (not statistically significant). These findings confirm that the biocomposite MPC+CS system remained structurally stable throughout the incubation period.

### 3.4. Mechanical Properties

The mechanical properties of the investigated cements are presented in Figure 8. The compressive modulus remained within a relatively narrow range of approx. 1.8–2.5 GPa for all formulations, and no statistically significant differences were observed between the tested groups (Figure 8B). In contrast, differences were observed in compressive strength (Figure 8A). The highest value, reaching approx. 37 MPa, was recorded for the MPC_B+1.5%CS_5.6%GP formulation. This value was significantly higher than those of MPC and MPC_B (as well MPC+CS, Table S2). The remaining CS/GP-modified formulations exhibited compressive strength values ranging from ~27–28 MPa and did not differ significantly. Representative mechanical curves are shown in Figure 8C. The MPC_B+1.5%CS_5.6%GP formulation reached the highest maximum stress among all tested groups. The stress–strain profiles of the remaining CS/GP-containing cements were generally comparable to the MPC_B, although slight differences in the compressive strength value were noticed. Overall, the results indicate that only the group with the higher CS concentration and lower GP content showed a noticeable improvement in mechanical performance, while the cements’ stiffness remained unaffected by the modifications.

### 3.5. Cytocompatibility

Figure 9 presents the cytocompatibility of the developed cements toward human osteoblasts after 3 days of direct culture. Cell viability remained above the cytotoxicity threshold defined by ISO 10993-5 [39] for all investigated formulations, confirming their cytocompatible character. Among the tested groups, MPC_B+1.0%CS_5.6%GP promoted the highest metabolic activity. Importantly, these results indicated that none of the applied modifications introduced any adverse biological effects.

## 4. Discussion

The development of dual-setting bone cements has attracted increasing attention as a strategy for overcoming the inherent limitations of conventional self-setting ceramics, particularly their limited cohesion and brittleness. By integrating a secondary polymer-mediated setting mechanism with cement crystallization, such systems provide an opportunity to improve handling characteristics while simultaneously influencing the physicochemical and mechanical properties of the resulting materials [40,41,42]. In the present study, a series of chitosan-based dual-setting magnesium phosphate cements was developed and systematically evaluated to establish the structure-property relationship governing their physicochemical, mechanical, and biological performance. The following sections discuss these relationships, with particular emphasis on the interactions between polymer network formation and cement crystallization and their impact on the final properties of the developed materials.

### 4.1. Selection of a Suitable Chitosan Derivative for Magnesium Phosphate Cement Modification

The first stage of this work focused on identifying a chitosan derivative capable of forming a cohesive, stable dual-setting MPC system suitable for bone applications. Although both CS and CMCS hydrogels have been widely investigated, their distinct physicochemical characteristics resulted in markedly different behavior when incorporated into the cement matrix. Native CS contains protonatable amino groups that enable pH-responsive gelation in the presence of β-glycerophosphate, promoting the formation of a continuous polymer network during cement setting [43]. In contrast, CMCS contains additional carboxymethyl substituents that increase water solubility and alter intermolecular interactions, resulting in a more dispersed polymer phase [32]. Consequently, the CS/GP system relied on simultaneous polymer gelation and MPC crystallization, whereas CMCS stabilization through Zn ions was expected to proceed primarily through their ionic interactions with carboxylate groups [30,44]. These fundamental differences in polymer network formation likely contributed to the distinct handling and cohesion characteristics observed for the investigated formulations.

The initial screening revealed that the choice of chitosan derivative had a considerably greater impact on handling characteristics and thermal behavior than on setting kinetics. Although both CS- and CMCS-containing formulations exhibited comparable setting times (Table 2A,B), their behavior during and after extrusion differed markedly. This observation suggests that the polymer contribution to dual-setting cement performance was governed primarily by the nature of the developing polymer network rather than by substantial changes in the rate of MPC crystallization itself.

In the CS-containing systems, GP should not be interpreted solely as a conventional thermosensitive gelation agent because the local chemical environment is rapidly altered by the MPC setting reaction. The initial acidic conditions associated with phosphate dissolution are progressively counterbalanced by MgO dissolution, which increases the pH and promotes CS chain deprotonation [45,46]. Under these conditions, GP contributes to polymer stabilization by buffering the evolving pH environment and supporting electrostatic and hydrogen-bonding interactions within the CS-rich phase [43]. At the same time, phosphate groups present in GP may participate in the ionic environment of the cement paste and interact with Mg-containing species [11], indicating that GP can influence both polymer network formation and the hydraulic reaction of MPC. Therefore, the improved cohesion observed for CS-containing cements likely resulted from the coupled evolution of CS network formation and MPC crystallization rather than from isolated GP-induced gelation alone.

In contrast, CMCS introduces negatively charged carboxymethyl groups capable of interacting with divalent ions. ZnCO_3_ was incorporated together with GDL to promote ionic stabilization of the CMCS phase; however, the resulting network appeared less effective at maintaining paste integrity under wet conditions (Figure 2). This may be related to the higher solubility and more dispersed character of CMCS [32], which can limit the formation of a continuous polymer-rich phase capable of reinforcing the fresh cement structure. Furthermore, Zn^2+^ ions released during acidification may not have acted exclusively as CMCS cross-linking centers, as they could also interact with phosphate species involved in MPC setting [47]. Such competing interactions may have reduced the efficiency of CMCS stabilization and contributed to the poorer cohesion observed for these formulations.

The different thermal behavior of the investigated systems further supports this interpretation. While CS/GP-modified cements maintained maximum reaction temperatures comparable to those of MPC_B, all CMCS-containing formulations exhibited significantly higher temperatures (Table 2). The lower temperature observed for the CS systems (as both CS and B were reported previously as potential retarders [14,48]) suggests that the combined effects of GP incorporation and progressive polymer network formation moderate the exothermic MPC setting reaction.

In contrast, the more pronounced exothermic response observed for the CMCS-based system may potentially be associated with the weaker stabilization of the CMCS phase and competition between Zn^2+^ and Mg^2+^ ions during cement formation. However, this interpretation remains hypothetical and requires further experimental verification. From a clinical perspective, this distinction is particularly relevant, as excessive heat generation during cement setting may adversely affect surrounding tissue and compromise the safety of injectable bone substitutes [49].

Taken together, these findings indicate that CS/GP provided a more favorable balance between setting behavior, thermal response, and paste cohesion than CMCS/Zn^2+^. Consequently, CS-based formulations were selected for subsequent investigations aimed at elucidating the structure-property relationships governing the physicochemical, mechanical, degradation, and biological performance of dual-setting MPC+CS systems.

### 4.2. Interplay Between Chitosan Gelation and MPC Setting Reaction

The setting behavior of MPC is governed by the dissolution of MgO and subsequent precipitation of k-struvite [45], whereas the CS/GP system undergoes thermosensitive gelation in response to changes in temperature and pH [50]. Therefore, the incorporation of CS hydrogel was expected to introduce an additional process occurring simultaneously with cement hardening. The prolonged setting time, moderated temperature rise (Table 2), and altered pH evolution (Figure 4) observed after modification indicate that the polymer phase interacted with the cement setting process.

The incorporation of the CS-GP system influenced the early-stage setting behavior of the investigated formulations, as reflected by the prolonged setting time, reduced maximum reaction temperature, and slower pH evolution compared to the unmodified MPC. Similar effects have been widely reported for polymer-modified MPC, in which polymeric additives act as setting retarders by increasing paste viscosity, restricting ion mobility, and partially hindering the dissolution of MgO particles and subsequent crystal growth [23,51,52]. In the present study, however, the observed effects are likely associated not only with the presence of chitosan itself, but also with the formation of a thermosensitive CS/GP hydrogel phase. Unlike conventional polymer solutions, hydrogel-forming systems can progressively develop a three-dimensional network during cement hardening, which may further influence mass transport and reaction kinetics within the setting cement [40]. However, our preliminary studies (Table S1) found that the CS addition alone was insufficient to adequately reduce the exothermic reaction associated with MPC setting, with the maximum reaction temperature remaining at ~43 °C. Therefore, borax was incorporated as an additional retarder o further control the hydration process and improve handling characteristics. Consequently, the observed changes in setting time, reaction temperature, and pH evolution should be attributed to the combined effect of B and the CS/GP system rather than to a single component. Nevertheless, all formulations exhibited a rapid increase in pH during the first minutes after mixing and ultimately reached similar final pH values. Collectively, these observations indicate that the CS/GP system acted primarily as a kinetic modifier of the setting process, delaying the early stages of cement hardening without altering the final reaction outcome.

The gradual increase in pH, together with the exothermic nature of the MPC reaction, likely created favorable conditions for thermosensitive CS gelation in the presence of GP. Previous studies have demonstrated that GP enables the formation of injectable chitosan solutions that undergo temperature- and pH-dependent gelation via a combination of electrostatic interactions, hydrogen bonding, and hydrophobic interactions [53,54]. Consequently, the MPC reaction may have facilitated the formation of a chitosan-rich network within the cement matrix. However, no direct evidence of such a network was obtained from FTIR analysis (Figure 5B), likely due to the relatively low CS content and the dominance of characteristic phosphate-related bands in the hardened cement spectra [48]. Similarly, SEM observations (Figure 3) did not reveal any distinct polymer-rich domains, suggesting that the CS phase was homogeneously dispersed throughout the cement matrix.

Interestingly, increasing the GP concentration from 5.6% to 8.0% resulted in only minor differences in setting time, reaction temperature, and pH evolution. This finding may suggest that the lower GP concentration was already sufficient to induce effective thermosensitive gelation, and that further GP addition had a limited effect on the overall setting behavior of the system.

Overall, the obtained results are consistent with the concept of a dual-setting system in which CS gelation and MPC crystallization proceed concurrently.

### 4.3. Structural Implications and Reinforcement Mechanisms in CS-Modified MPC

The structural characterization demonstrated that the incorporation of CS hydrogel had only a limited influence on the final architecture of the hardened MPC matrix. Neither SEM observations (Figure 3) nor phase and chemical analyses (Figure 5, Table 3) revealed substantial differences between the modified formulation and the reference cements.

The XRD patterns remained dominated by characteristic k-struvite and residual MgO reflections, while FTIR spectra showed comparable absorption profiles for all investigated groups. These findings suggest that CS hydrogel incorporation did not alter the fundamental acid-base reaction responsible for MPC setting and did not lead to the formation of additional crystalline phases. Instead, the polymer most likely acted as a physical modifier embedded within the cement matrix, influencing microstructural organization rather than its chemical composition. Similar observations have been reported for polysaccharide-modified MPC, where polymer additives primarily affect matrix cohesion and microstructure while preserving the characteristic cement chemistry [25,52].

Likewise, SEM examination revealed no pronounced morphological differences between the tested formulations. The absence of distinct polymer-rich regions is not unexpected, given the relatively low polymer content and chitosan’s predominantly amorphous nature [55]. Following gelation and cement hardening, the CS phase was likely distributed as a thin, interpenetrating network within the inorganic matrix, making its direct visualization with conventional SEM difficult. Similar observations have been reported for chitosan-containing phosphate cement [23,24,56].

Although the overall microstructure remained largely unchanged, differences were observed in the porosity results (Figure 6). A statistically significant increase in total open porosity was detected only for the MPC_B+1.5%CS+8.0%GP formulation. This effect may be associated with the elevated GP concentration, affecting the kinetics of simultaneous CS gelation and MPC crystallization. As GP participates in the thermosensitive CS gelation, its higher content may promote the formation of more developed polymer-rich phases within the cement paste. Such domains could locally restrict ion diffusion and interfere with homogeneous crystal growth, leading to less-dense packing of the organic matrix and a higher volume of interconnected pores. The absence of similar changes in the MPC_B+1.5%CS_5.6%GP formulation suggests that this effect becomes relevant only above a certain GP concentration threshold. It is worth noting that previous studies on polymer-modified and dual-setting cements have generally reported either a reduction or no significant change in porosity following polymer incorporation [19,24,26,57]. In most cases, the polymer phase was proposed to occupy intercrystalline spaces and partially fill preexisting voids, resulting in a denser or structurally preserved microstructure. Therefore, the increased porosity observed in this study appears to be associated not with the presence of CS itself, but rather with the elevated GP content and its influence on the balance between hydrogel formation and cement crystallization during setting.

Interestingly, despite the observed increase in porosity of MPC_B+1.5%CS+8.0%GP, no significant differences in compressive modulus were detected among the investigated formulations (Figure 8). This observation is consistent with the XRD and FTIR results (Figure 5), which indicated no substantial changes in the phase composition or chemical structure of the hardened cements. The preservation of the characteristic MPC phases likely maintained the overall rigidity of the cement matrix, while the observed changes in porosity were insufficient to significantly affect its elastic response. In contrast, the compressive strength results suggest that the mechanical performance of the CS/GP-modified cements was strongly dependent on the composition of the polymer phase. It is noteworthy that a previous study on CS-modified MPC without an additional cross-linking agent (such as GP) reported no significant change in compressive strength [24]. In the present study, however, MPC_B+1.5%CS_5.6%GP exhibited significantly higher compressive strength than the control formulations (MPC, MPC_B, and MPC+CS). This finding suggests that incorporating GP at an appropriate concentration may promote the formation of a polymer network that positively influences the load-bearing behavior of the cement—as observed in Figure 8C. The absence of a similar strengthening effect in the remaining CS/GP-containing formulations indicates that the relation between hydrogel composition and mechanical performance is not straightforward. In particular, increasing the GP concentration from 5.6% to 8.0% did not yield further improvement in compressive strength. This observation may be associated with the increased porosity observed in this group (Figure 6), which could partially offset any beneficial effect of the polymer phase [58]. Increased porosity generally reduces the effective load-bearing cross-section of cementitious materials and introduces structural discontinuities that may act as stress concentrators, facilitating crack initiation and propagation [59,60]. Accordingly, the mechanical performance of the developed dual-setting cements appears to reflect a balance between polymer-mediated reinforcement and the pore structure generated during simultaneous hydrogel formation and cement crystallization. This may explain why MPC_B+1.5%CS_5.6%GP exhibited the highest compressive strength, whereas further increasing the GP concentration provided no additional mechanical benefit. Similar positive effects for polymer-mediated reinforcement have been previously reported for polysaccharide-modified cements, including CMCS-containing formulations [18,19,26]. The improved mechanical performance may be attributed to the formation of a more homogeneous CS-based network that may facilitate stress distribution throughout the cement matrix and reduce the susceptibility to microcrack propagation under compressive loading [61,62].

Collectively, these findings indicate that CS/GP modification did not fundamentally alter the phase composition, chemical structure, morphology, or stiffness of the hardened MPC. Instead, the results suggest that the material’s mechanical response was sensitive to the specific CS/GP ratio and the resulting pore structure. This relationship may also be relevant to degradation behavior, since water-accessible porosity determines the pathways available for fluid penetration into the cement matrix and may therefore influence subsequent dissolution and mass loss during aqueous incubation [8,63].

### 4.4. Degradation, Cytocompatibility and Medical Potential of the Developed Composite Cements

The degradation behavior of bone cements is an important factor influencing their long-term stability and in vivo performance [64]. All investigated formulations exhibited relatively low mass loss after 28 days of incubation in PBS (Figure 7). Although the incorporation of CS at a higher concentration resulted in a slight increase in mass loss, the difference was not statistically significant. These findings indicate that the CS/GP modification did not adversely affect the structural stability of the cement matrix. When considered together with the porosity results, the degradation data further indicate that the relationship between pore structure and mass loss was not directly proportional across the investigated formulations. In principle, a higher fraction of water-accessible pores may facilitate penetration of the aqueous medium into the cement matrix, increase the solid–liquid interfacial area, and thereby promote dissolution of the cement phase [9,65]. However, although MPC_B+1.5%CS+8.0%GP exhibited significantly higher open porosity, this did not translate into a statistically significant increase in mass loss after 28 days. This suggests that, within the investigated range, the differences in pore accessibility were insufficient to substantially accelerate bulk degradation. Instead, degradation was likely governed by the combined effects of pore accessibility, dissolution of the magnesium phosphate matrix, and stabilization of the CS phase within the inorganic network. The relatively low degradation rates observed for all formulations are consistent with the preserved phase composition (Figure 5) and microstructure (Figure 3) of the hardened cements. Furthermore, results suggest that CS remained effectively integrated within the cement matrix rather than undergoing rapid dissolution. This observation is particularly noteworthy, given that standalone CS hydrogels exhibit considerably greater mass loss under aqueous conditions [66,67]. In the present system, however, the polymer phase was likely confined within the inorganic cement network, which may have limited its release into the surrounding medium.

From a mechanical perspective, progressive dissolution of the cement matrix and the associated development or interconnection of water-accessible pores may gradually reduce the effective load-bearing fraction of the material during prolonged aqueous exposure. Thus, porosity, mass loss, and compressive performance should be regarded as interrelated parameters rather than independent characteristics of the cement [68,69]. Nevertheless, because compressive strength after prolonged PBS incubation was not evaluated in the present study, the evolution of mechanical properties during degradation cannot be directly inferred from the mass-loss results and warrants further investigation. It should also be noted that degradation in PBS primarily reflects dissolution and leaching processes [70]. As PBS represents a simplified, cell-free environment, the relatively low mass loss observed in the present study should not be directly extrapolated to in vivo degradation. Indeed, these results may underestimate the degradation rate under physiological conditions, where dynamic fluid exchange, enzymatic activity, cellular interactions, and continuous tissue remodeling further contribute to biomaterial degradation.

The cytocompatibility results further confirmed the suitability of the developed materials for bone-related applications. All formulations maintained cell viability above the 70% threshold specified in ISO 10993-5 (Figure 9), demonstrating the absence of cytotoxic effects associated with the investigated modification. This finding was not unexpected, as all constituents of the developed system, including MPC, CS, GP, and B, have been extensively investigated previously and are generally regarded as cytocompatible materials for biomedical applications. Numerous studies, both in vitro and in vivo, have demonstrated their favorable biological responses and suitability for bone regeneration [8,43,71,72]. Importantly, a direct-contact culture was performed over 3 days, confirming not only osteoblast survival but also their ability to adhere to the cement surface and maintain metabolic activity throughout the incubation period [73]. This observation clearly suggests that the incorporation of the CS/GP system did not impair cell-material interactions that are essential for subsequent tissue regeneration.

From a practical medical perspective, the combination of good cytocompatibility, limited degradation, and the improved extrusion behavior and cohesion observed for the CS-containing formulations supports their potential use as injectable bone substitutes. In particular, maintaining structural stability while preserving favorable handling properties is advantageous for the clinical application of injectable bone cements [74], where controlled placement and resistance to washout are essential for successful defect filling and tissue regeneration. Among the investigated formulations, MPC_B+1.5%CS_5.6%GP demonstrated the most balanced performance, combining improved handling characteristics and enhanced compressive strength, making it the most promising candidate for further biological evaluation and potential bone regeneration applications.

### 4.5. Study Limitations and Future Research Directions

While the present study provides a comprehensive physicochemical, mechanical and biological evaluation of MPC+CS/GP cements, several aspects warrant further investigation. First, the improvement in injectability was assessed primarily through qualitative observations. Future studies could complement these findings with quantitative injectability measurements using a universal testing machine (equipped with dedicated extrusion fixtures), enabling a more detailed assessment of extrusion forces and handling performance under clinically relevant conditions. Additionally, dynamic oscillatory rheological measurements should be performed to quantitatively characterize the viscoelastic behavior of the cement pastes and monitor its evolution during setting. Such measurements could provide further insight into the effects of the incorporated hydrogel systems on paste cohesion and the development of the composite structure. Another limitation of the present study is that the formation of the cross-linked CS network within the MPC matrix could not be directly confirmed. The FTIR spectra of the composite cements were dominated by signals originating from the inorganic MPC phase, limiting the identification of spectral changes specifically associated with CS gelation. Therefore, although the setting reaction of the MPC phase was confirmed by XRD and the observed changes in paste cohesion and material properties support the contribution of the hydrogel component, the proposed dual-setting mechanism should be regarded as indirectly supported rather than directly structurally verified.

In addition, although the developed formulations demonstrated favorable cytocompatibility toward human osteoblasts, further biological investigations would provide a more comprehensive understanding of their regenerative potential and enable the identification of formulations with the most favorable biological performance. In particular, studies focusing on osteogenic differentiation, matrix mineralization, and long-term cell-material interactions (possibly with primary cells) may help elucidate the influence of the CS/GP system on bone-forming processes. Ultimately, in vivo evaluation will be necessary to assess material degradation, tissue response, and bone regeneration under physiological conditions. As a final step, the most promising CS/GP-modified MPC formulations should be evaluated in a relevant bone defect model, with particular attention to osseointegration and their capacity to support new bone formation over time. Furthermore, the present results suggest that the CS/GP modification primarily influenced the setting behavior and selected functional properties of MPC without substantially altering its phase composition and morphology. Therefore, future studies employing complementary characterization techniques, such as Raman spectroscopy or micro-computed tomography, may provide additional insights into the spatial distribution of the polymer phase and its interactions with the inorganic cement matrix.

Overall, the findings of this works identify CS/GP-modified MPC as a promising platform for further optimization. Future research may focus on tailoring the polymer composition and gelation conditions to further improve handling characteristics. In parallel, optimizing cement-related parameters, such as the Mg/P molar ratio and P/L ratio, may offer additional opportunities to shorten setting time and reduce unreacted MgO in the hardened cement matrix. Such modifications could further enhance the clinical applicability of the developed dual-setting composite system.

## 5. Conclusions

This study demonstrates a novel dual-setting strategy for magnesium phosphate cements (MPCs) achieved by combining borax (B) with a thermosensitive chitosan/β-glycerophosphate (CS/GP) hydrogel. While B primarily acted as a hydration modifier controlling the rapid acid-base reaction of MPC, the CS/GP phase introduced an additional gelation mechanism that further influenced the setting behavior and handling characteristics of the cement. The synergistic action of both components enabled the development of injectable biocomposite cements with improved processing features while preserving the intrinsic properties of the inorganic matrix.

The incorporation of the B/CS/GP system effectively prolonged the setting time, reduced the maximum reaction temperature, and slightly modified pH evolution during cement hardening. Importantly, these improvements were achieved without substantial alterations in phase composition, chemical structure, or microstructure—as confirmed by XRD, FTIR, and SEM analyses. Furthermore, all developed formulations exhibited a low degradation rate (~6–8% mass loss after 28 days) and maintained favorable cytocompatibility toward human osteoblasts (comparable with tissue culture polystyrene).

Among the investigated compositions, the MPC cement containing 0.5% borax, 1.5% chitosan, and 5.6% β-glycerophosphate emerged as the most balanced system. This formulation successfully combined improved qualitative injectability and cohesion with the highest compressive strength (~37 MPa), with slightly increased stiffness (~2.5 GPa), structural stability, and a favorable biological response. Collectively, these findings demonstrate that the selected formulation provides an advantageous balance of handling, physicochemical, mechanical, and biological properties.

Overall, this work establishes thermosensitive CS/GP gelation as a versatile and promising approach for designing dual-setting MPCs. The presented strategy provides a foundation for the development of clinically relevant injectable bone substitutes, in which setting behavior and handling characteristics can be tailored without compromising the fundamental performance of the cement matrix.

## Figures and Tables

**Figure 1:**
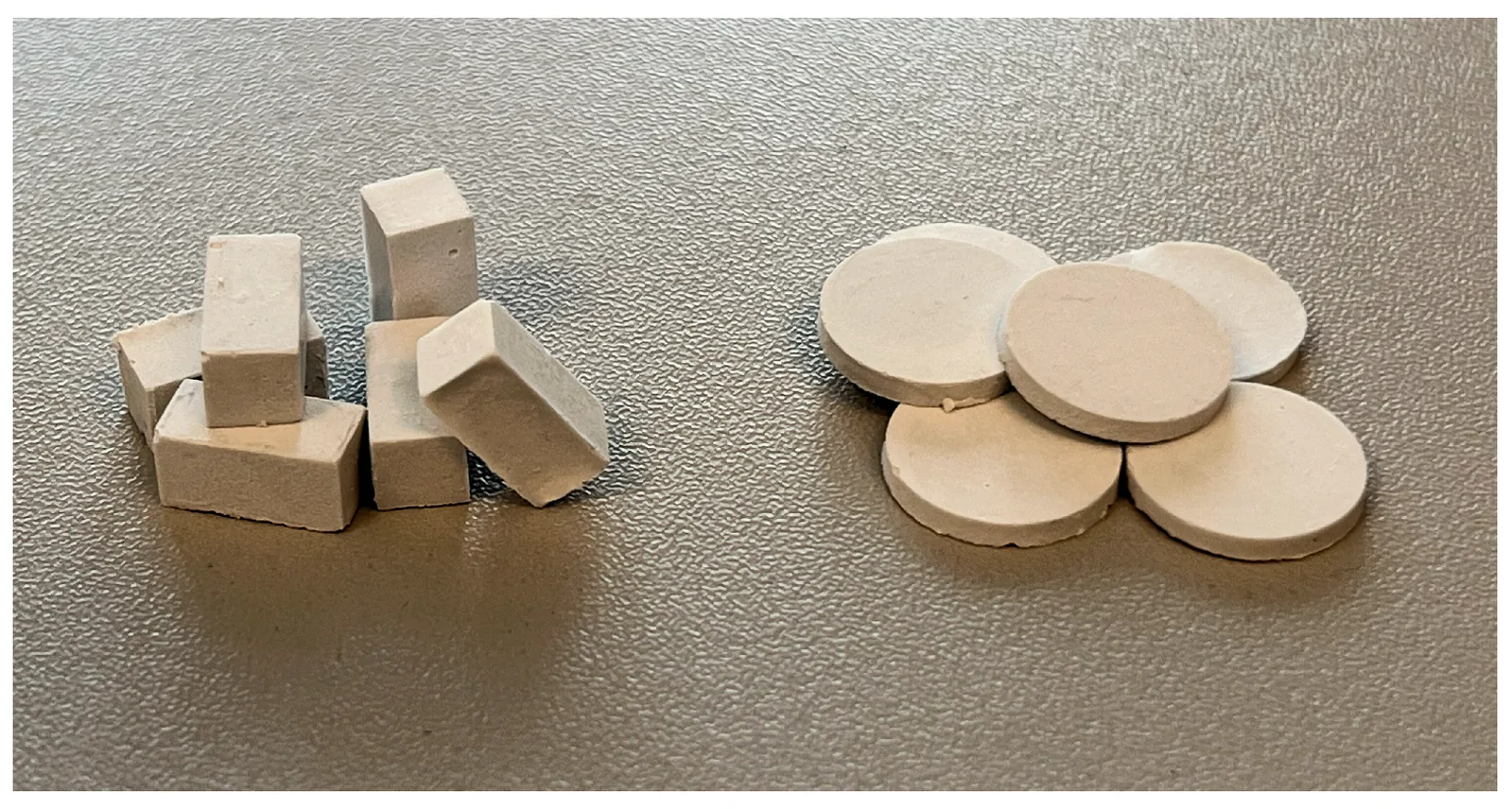
Representative photograph of the tested bone cement specimens after 24 h of curing.

**Figure 2:**
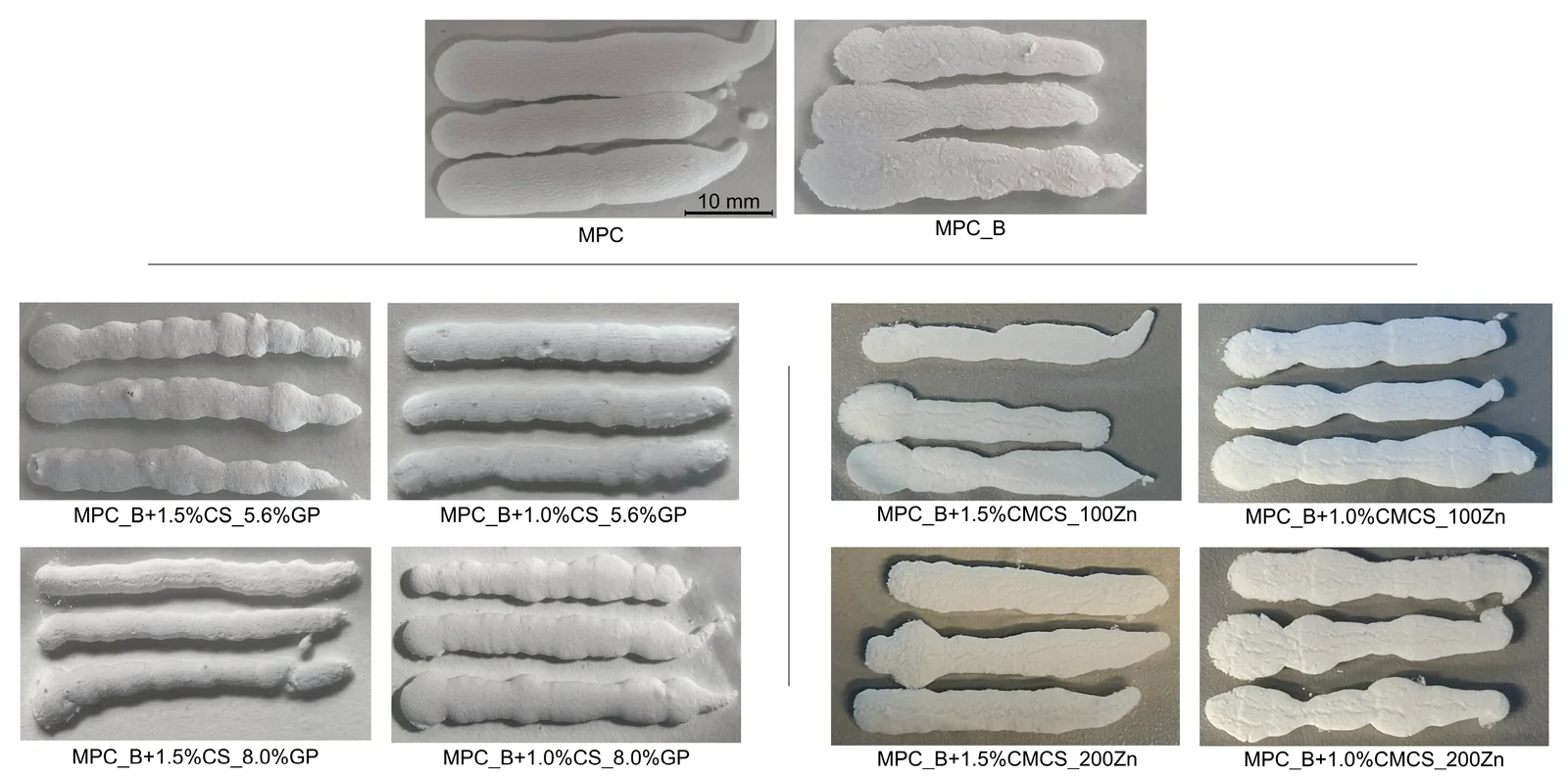
Qualitative evaluation of injectability and cohesion of the developed bone cements containing CS- and CMCS-based hydrogel under wet conditions.

**Figure 3:**
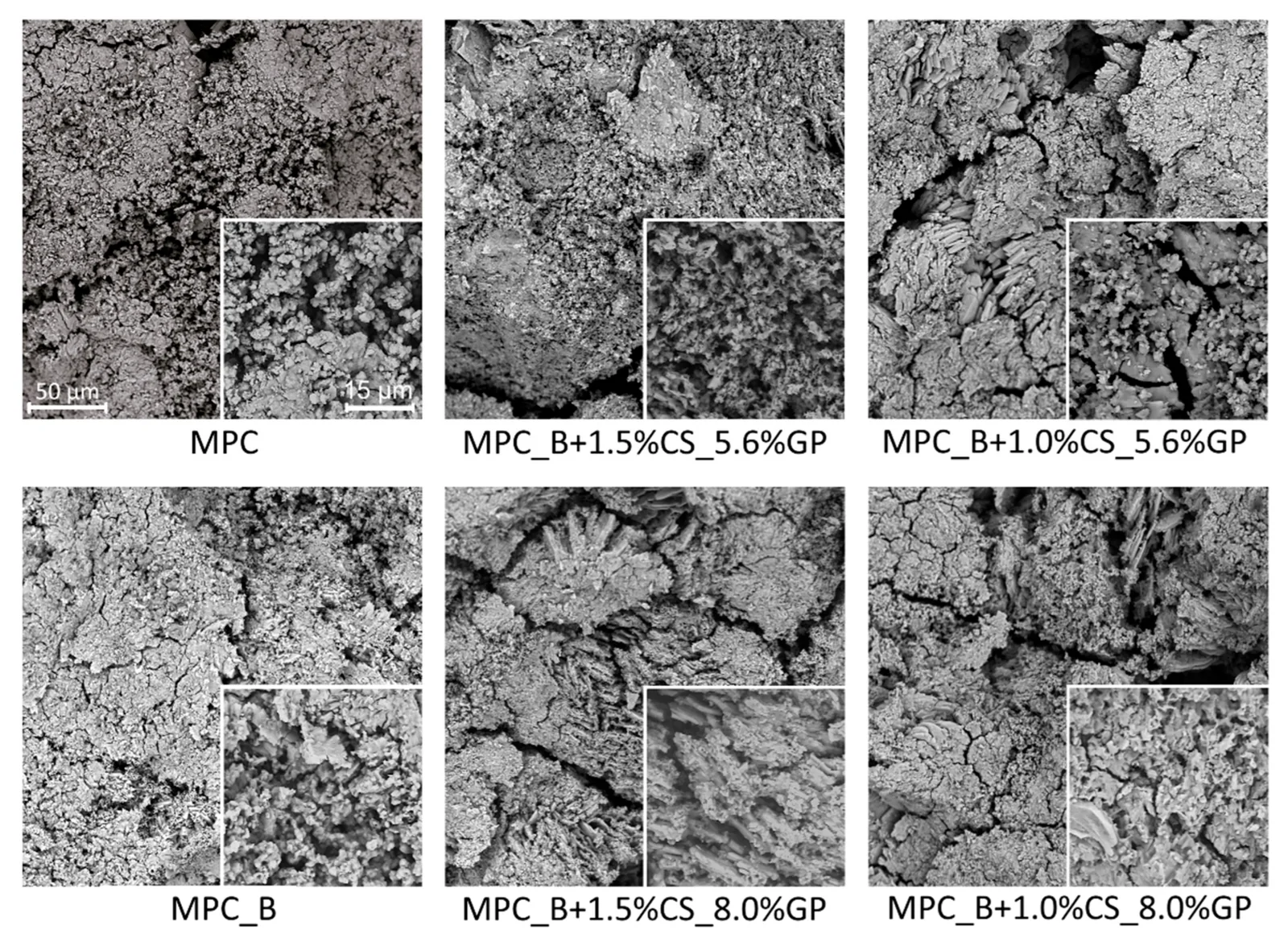
Representative SEM micrographs of the surfaces of the developed bone cements after curing (fractured cross-section), recorded at 500× and 2000× magnifications. The images represent three independent analyses.

**Figure 4:**
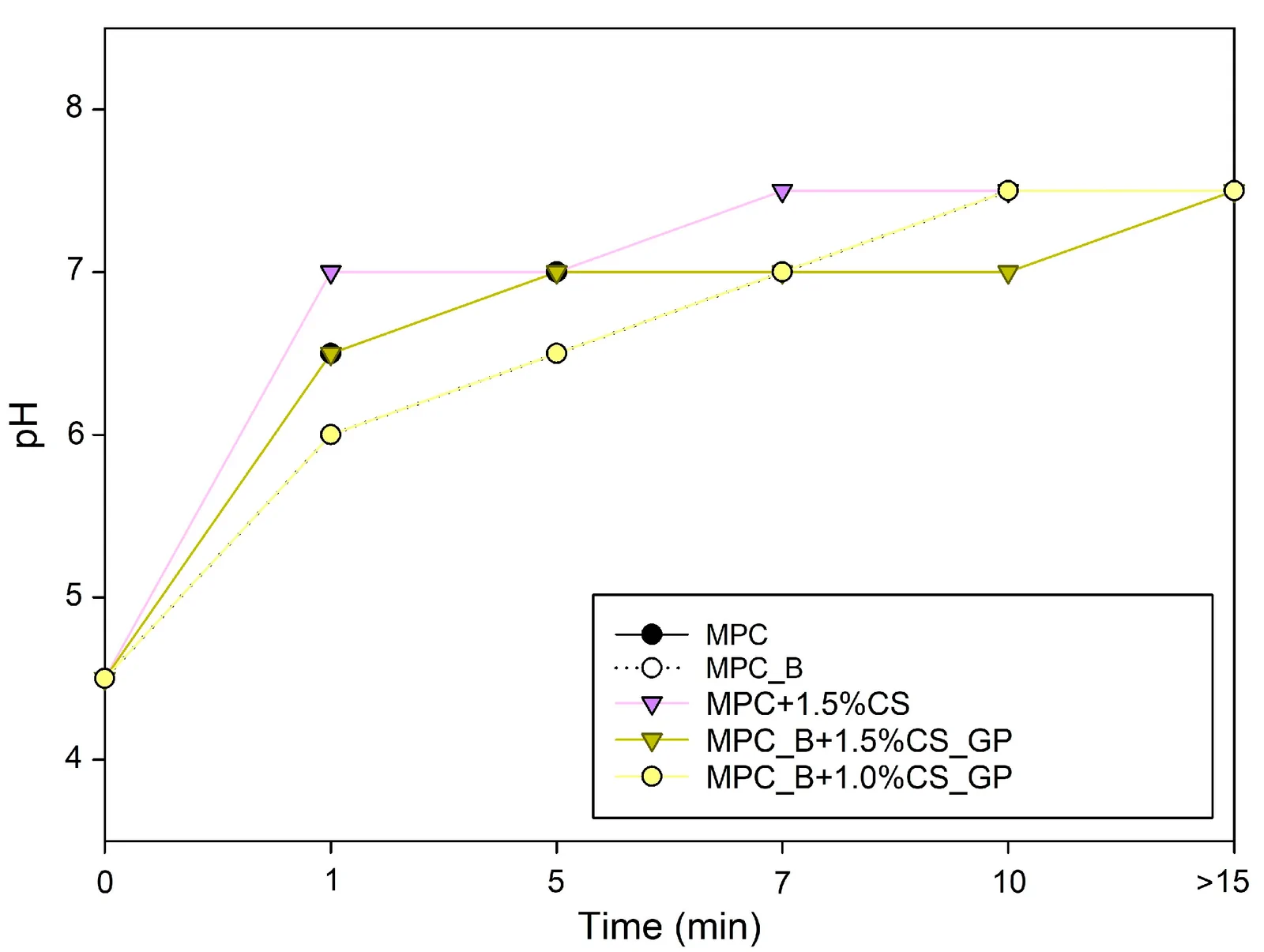
pH evolution of the investigated bone cement formulations during the initial setting period (*n* = 3). GP denotes both tested formulations: 5.6% GP and 8.0% GP.

**Figure 5:**
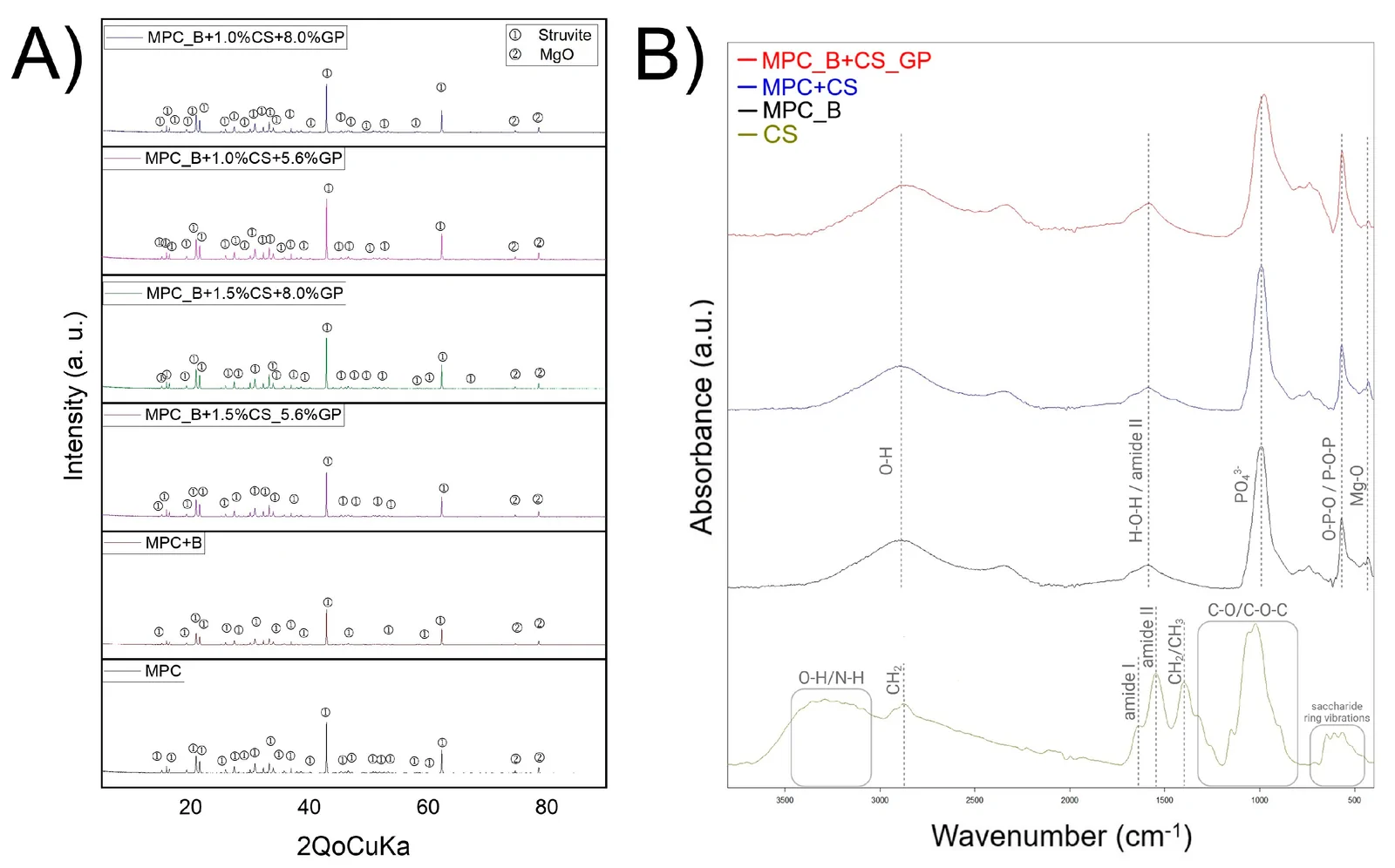
XRD patterns (**A**) and FTIR spectra (**B**) of the tested bone cements after curing. (**A**) The characteristic reflections are marked as follows: (1) k-struvite, (2) MgO, and (3) apatite. (**B**) MPC_B_CS and MPC_B_CS+GP are representative of both CS/GP concentrations.

**Figure 6:**
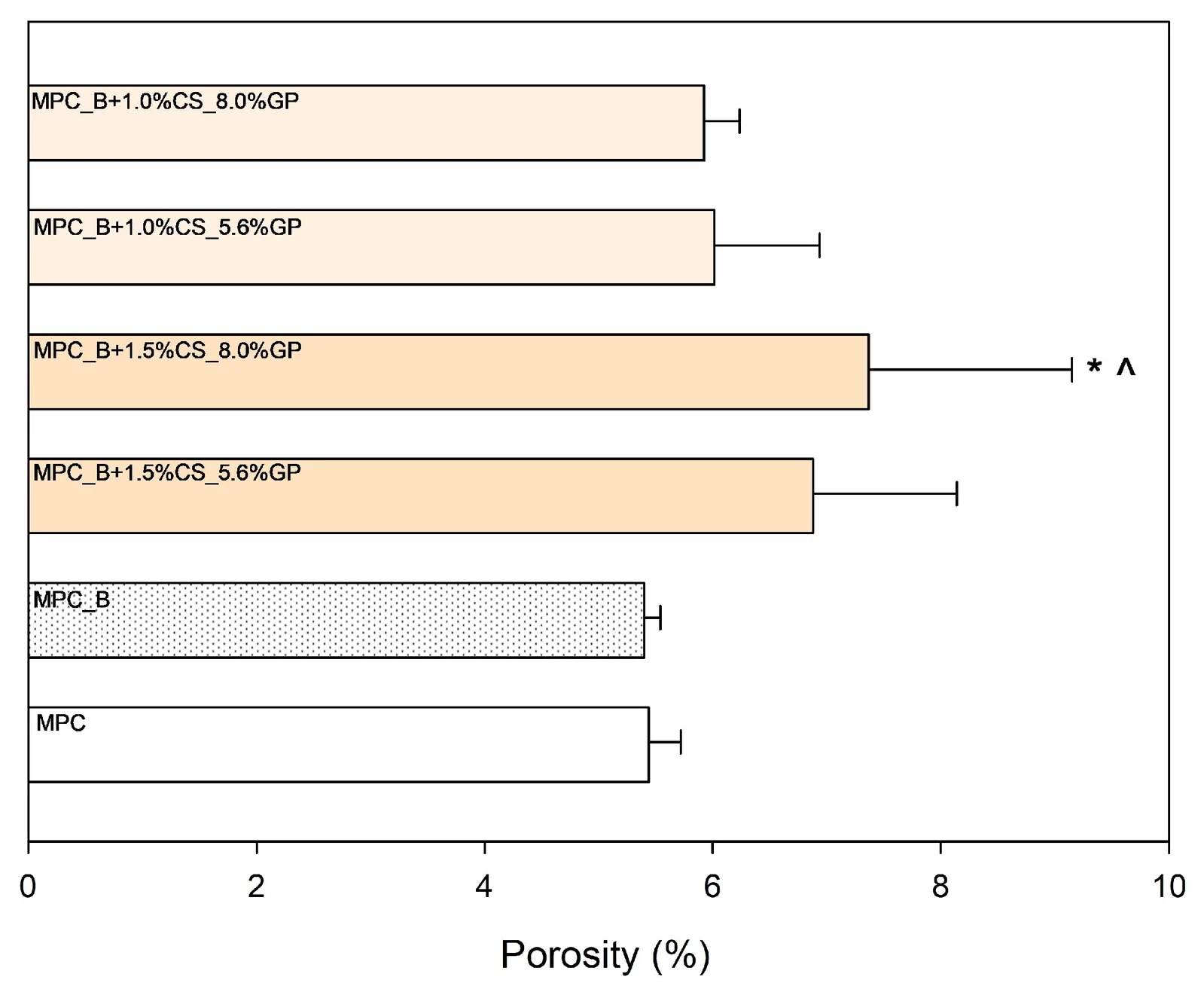
Open porosity of the tested bone cements (*n* = 4; data are expressed as the mean ± SD; * statistically significant difference compared with MPC (*p* < 0.05); ^ statistically significant difference compared with MPC_B (*p* < 0.05). No statistically significant difference vs. significant difference between GP concentrations, wherever the corresponding symbol is not displayed.

**Figure 7:**
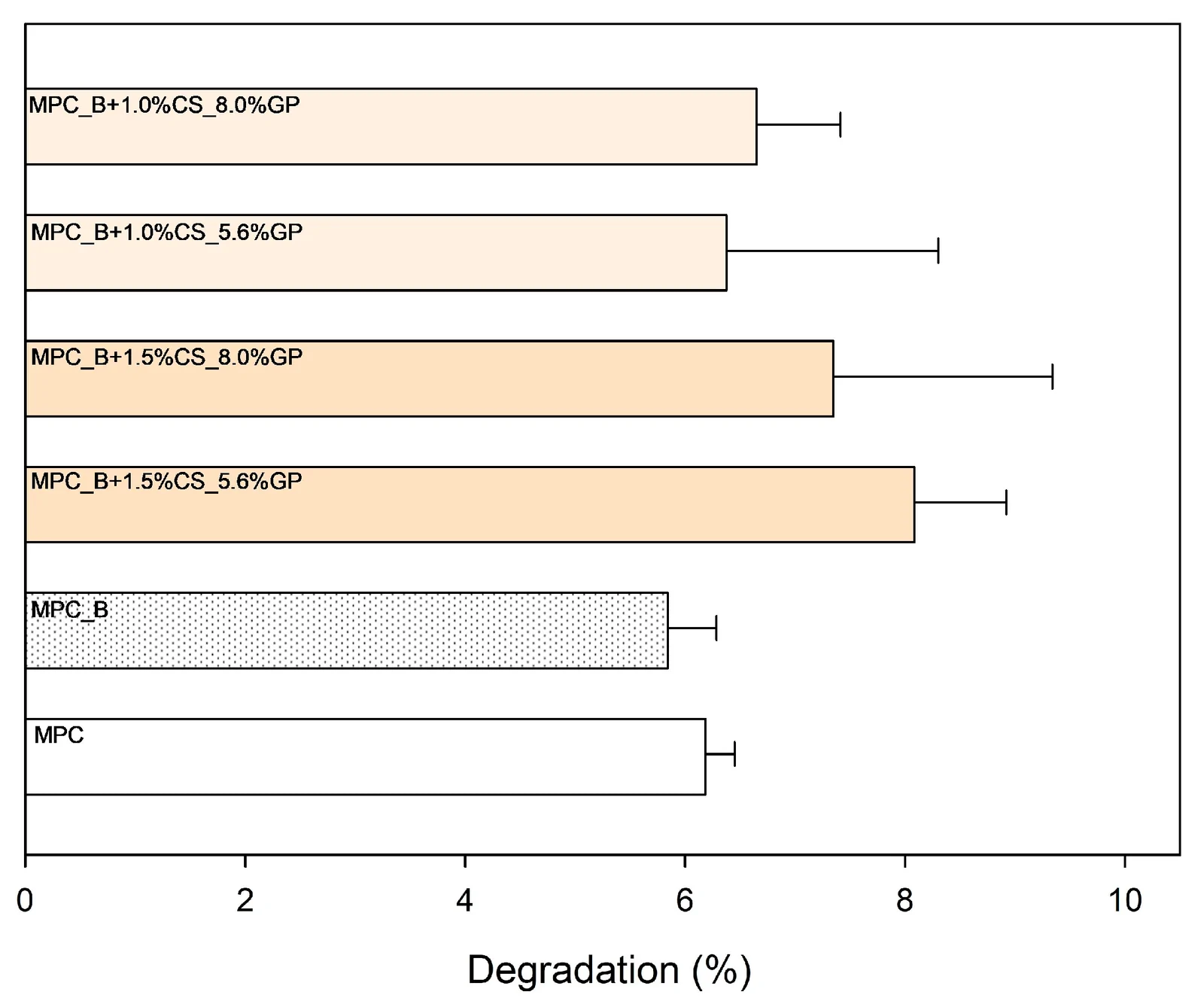
Mass loss of the tested bone cements after 28 days of incubation in PBS (*n* = 4; data are expressed as the mean ± SD; No statistically significant difference compared with MPC, wherever the corresponding symbol is not displayed; No statistically significant difference compared with MPC_B, wherever the corresponding symbol is not displayed; No statistically significant difference vs. GP concentrations, wherever the corresponding symbol is not displayed.

**Figure 8:**
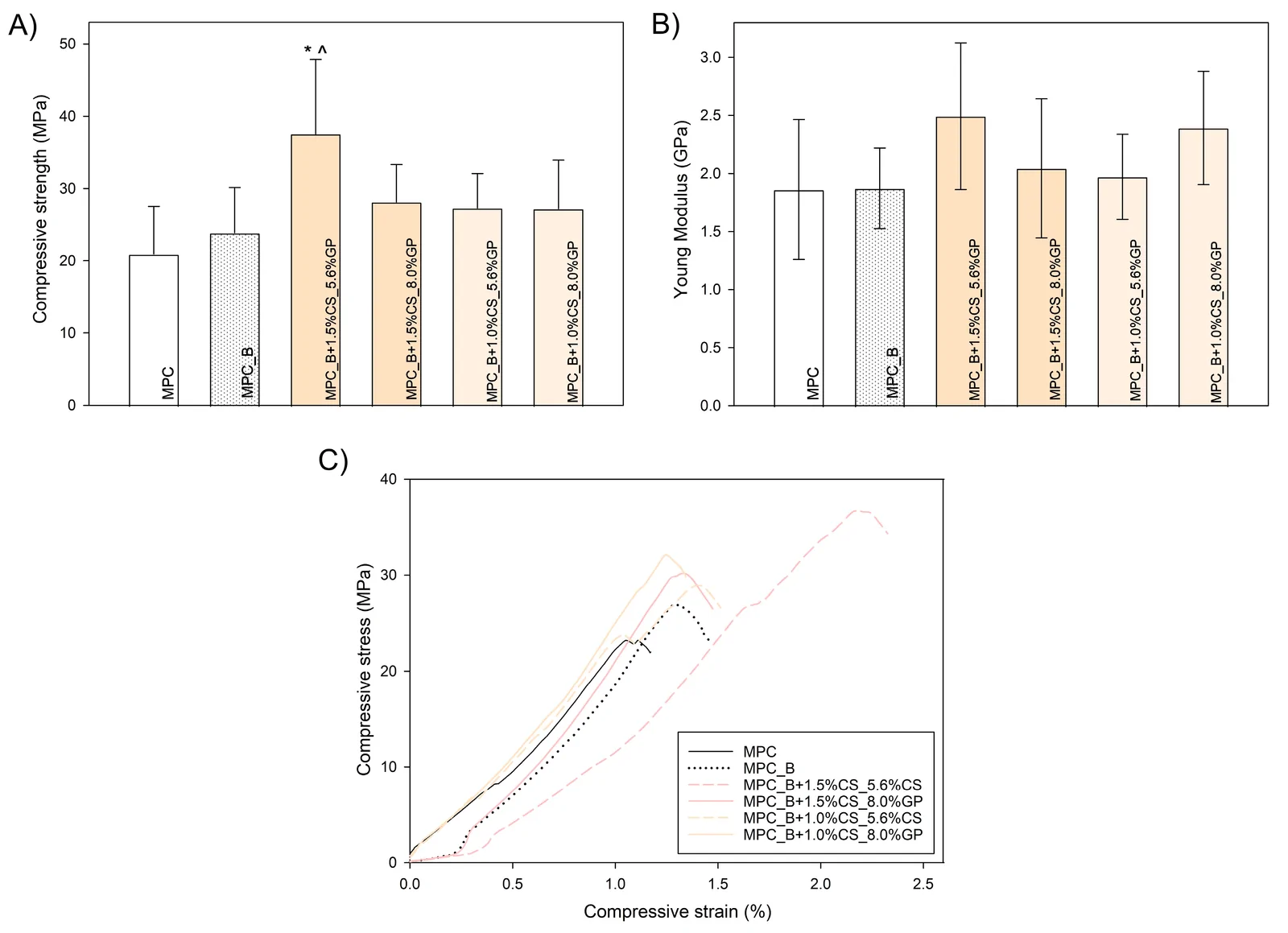
Mechanical properties of the tested bone cements (*n* = 7): (**A**) compressive strength (σc) and (**B**) Compressive modulus (Ec). Data are expressed as the mean ± SD. * Statistically significant difference compared with MPC (*p* < 0.05); ^ statistically significant difference compared with MPC_B (*p* < 0.05); No statistically significant difference vs. GP concentrations, wherever the corresponding symbol is not displayed. (**C**) representative mechanical curves.

**Figure 9:**
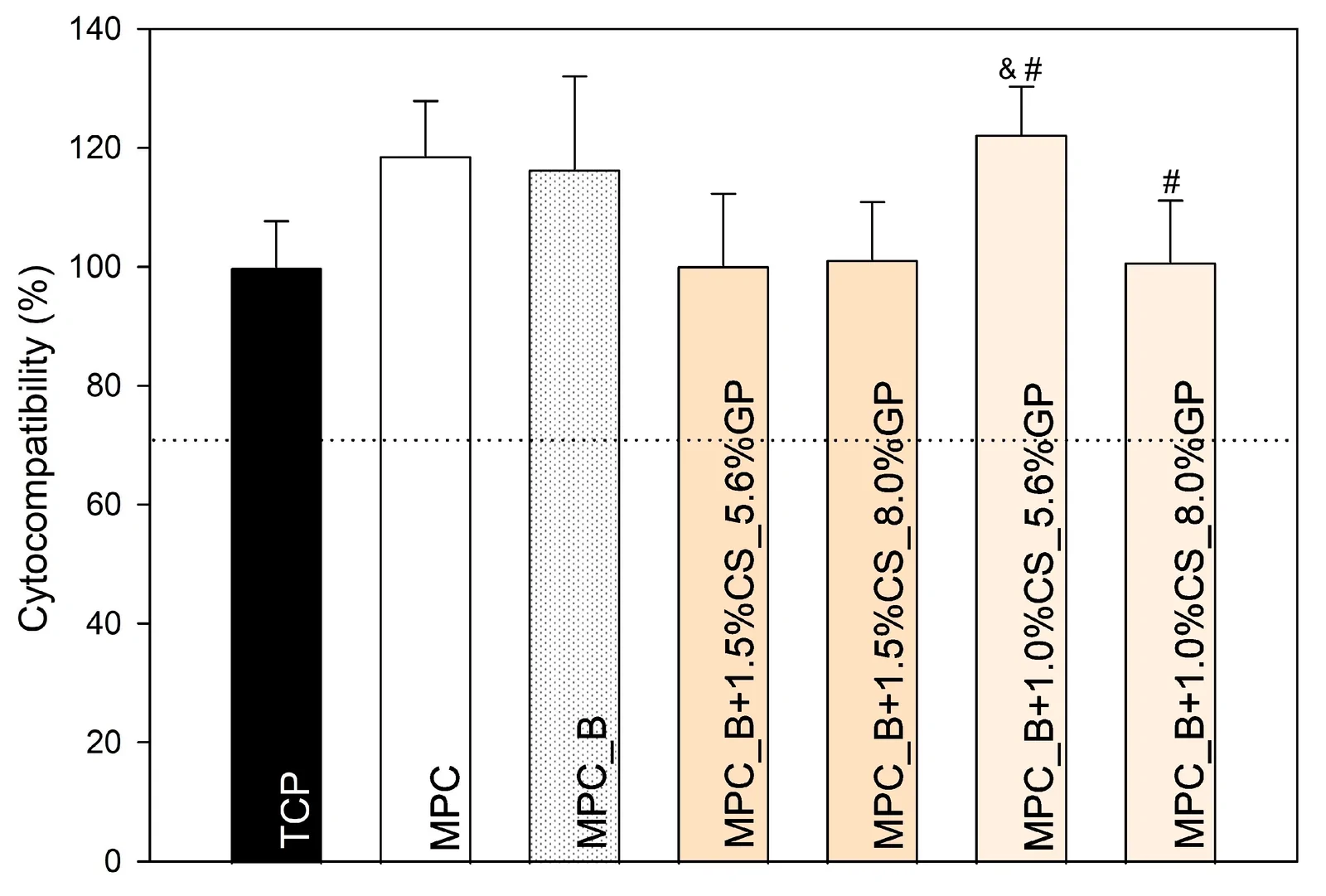
Cytocompatibility of the tested bone cements toward hFOB 1.19 cells after 3 days of direct contact with the material surfaces (*n* = 4). Data are expressed as the mean ± SD. The dashed line indicates the threshold for non-cytotoxicity according to ISO 10993-5. No statistically significant difference vs. MPC, wherever the corresponding symbol is not displayed; No statistically significant difference vs. MPC_B, wherever the corresponding symbol is not displayed; & statistically significant difference compared with TCP (*p* < 0.05); # statistically significant difference between GP concentrations (*p* < 0.05).

**Table 1:** Composition of the investigated dual-setting magnesium phosphate–chitosan cement formulations (“–” indicates that the corresponding component was not added to the formulation).

Formulation *	CS (%)	CMCS (%)	GP (%)	ZnCO_3_ (mM) ^	GDL (mM) ^	B (%)
MPC	–	–	–	–	–	–
MPC_B	–	–	–	–	–	0.5
MPC_B+1.5%CS_5.6%GP	1.5	–	5.6	–	–	0.5
MPC_B+1.5%CS_8.0%GP	1.5	–	8.0	–	–	0.5
MPC_B+1.0%CS_5.6%GP	1.0	–	5.6	–	–	0.5
MPC_B+1.0%CS_8.0%GP	1.0	–	8.0	–	–	0.5
MPC_B+1.5%CMCS+100Zn	–	1.5	–	100	14.03	0.5
MPC_B+1.5%CMCS+200Zn	–	1.5	–	200	28.06	0.5
MPC_B+1.0%CMCS+100Zn	–	1.0	–	100	14.03	0.5
MPC_B+1.0%CMCS+200Zn	–	1.0	–	200	28.06	0.5

* All formulations were prepared using a fixed Mg/P molar ratio of 4:1 and a P/L ratio of 2.5 g/mL. ^ Fixed GDL-to-Zn^2+^ molar ratio was used based on previous studies.

**Table 2:** (**A**) Effect of CS incorporation on the setting time and maximum reaction temperature of MPC (*n* = 3, data are expressed as the mean ± SD; * statistically significant difference as compared to MPC (*p* < 0.05), ^ statistically significant difference as compared to MPC_B (*p* < 0.05), ^#^ statistically significant difference between GP concentrations (*p* < 0.05)). (**B**) Effect of CMCS incorporation on the setting time and maximum reaction temperature of MPC (*n* = 3, data are expressed as the mean ± SD; * statistically significant difference as compared to MPC (*p* < 0.05), ^ statistically significant difference as compared to MPC_B (*p* < 0.05), ^#^ statistically significant difference between Zn concentrations (*p* < 0.05)).

(**A**)		
**Cement**	**Setting Time (min)**	**Reaction Temperature (°C)**
MPC	16.00 ± 2.47	55.53 ± 1.45
MPC_B	19.50 ± 0.87	41.23 ± 2.59
MPC_B+1.5%CS_5.6%GP	22.67 ± 2.08 *	39.10 ± 1.57 *
MPC_B+1.5%CS_8.0%GP	22.67 ± 2.62 *	40.33 ± 2.58 *
MPC_B+1.0%CS_5.6%GP	21.33 ± 1.89 *^#^	39.73 ± 1.23 *
MPC_B+1.0%CS_8.0%GP	25.50 ± 0.50 *^^#^	37.90 ± 3.55 *
(**B**)		
**Cement**	**Setting Time (min)**	**Reaction Temperature (°C)**
MPC	16.00 ± 2.47	55.53 ± 1.45
MPC_B	19.50 ± 0.87	41.23 ± 2.59
MPC_B+1.5%CMCS+100Zn	20.88 ± 1.49 ^#^	50.75 ± 1.53 *^
MPC_B+1.5%CMCS+200Zn	25.75 ± 1.32 *^^#^	49.58 ± 2.26 *^
MPC_B+1.0%CMCS+100Zn	22.13 ± 1.89 *	48.95 ± 2.46 *^
MPC_B+1.0%CMCS+200Zn	22.38 ± 1.60 *	52.23 ± 1.07 ^

**Table 3:** Quantitative phase composition of the tested bone cements determined by Rietveld refinement of XRD patterns (*n* = 3; all refinement estimated SD values ± 5.0%).

Cement	k-Struvite (%)	MgO (%)
MPC	60.6	39.4
MPC_B	60.7	39.3
MPC_B+1.5%CS_5.6%GP	61.1	38.9
MPC_B+1.5%CS_8.0%GP	61.1	38.9
MPC_B+1.0%CS_5.6%GP	60.8	39.2
MPC_B+1.0%CS_8.0%GP	61.2	38.8

## Data Availability

The original contributions presented in this study are included in the article/Supplementary Materials. Further inquiries can be directed to the corresponding author.

## Supplementary Materials

The following supporting information can be downloaded at: https://www.mdpi.com/article/10.3390/jfb17100481/s1, Table S1: Effect of CS polymer incorporation without a gelation agent on the setting time and maximum reaction temperature of MPC in preliminary studies; Table S2: Effect of CS polymer incorporation without a gelation agent on mechanical properties of MPC.

## Abbreviations

The following abbreviations are used in this manuscript:

ANOVA	Analysis of variance
B	Borax
BC	Bone cement(s)
CaP	Calcium phosphate(s)
CPC	Calcium phosphate cement(s)
CS	Chitosan
Ec	Compressive modulus
FTIR	Fourier-transform infrared spectroscopy
GP	β-glycerophosphate
hFOB	Human osteoblast cell line
ICSD	Inorganic Crystal Structure Database
ISO	International Organization for Standardization
Mg/P	Magnesium-to-phosphate
MPC	Magnesium phosphate cement
MTT	Thiazolyl blue tetrazolium bromide
PBS	Phosphate-buffered saline
PMMA	Poly(methyl methacrylate) cement(s)
P/L	Powder-to-liquid
SEM	Scanning electron microscopy
SD	Standard deviation
TCP	Tissue culture polystyrene
XRD	X-ray diffraction
σc	Compressive strength
