# Supplementary material for: Dual-Setting Magnesium Phosphate Bone Cement with Improved Handling Through the Integration of Chitosan-Derived Hydrogels
Source: J Funct Biomater. 2026 Sep 22;17(10):481. doi: 10.3390/jfb17100481 (PMC13706006; doi:10.3390/jfb17100481)
Supplement: Supplementary file 1 [file jfb-17-00481-s001.zip › jfb-4527034-supplementary.pdf]

Journal of
Functional Biomaterials

MDPI

Supplementary Information

Preliminary studies were conducted to evaluate whether the CS polymer alone could sufficiently moderate the exothermic reaction associated with MPC setting (Table S1).

Table S1. Effect of CS polymer incorporation without a gelation agent on the setting time and maximum reaction temperature of MPC in preliminary studies ($n = 3$, data are expressed as the mean ± SD; * statistically significant difference as compared to MPC ($p < 0.05$)).

Cement	Setting time (min)	Reaction temperature (°C)
MPC	16.00 ± 2.47	55.53 ± 1.45
MPC+1.5%CS	23.50 ± 1.45 *	43.37 ± 3.89 *

Additional studies were conducted to evaluate the effect of CS polymer alone, without a gelation agent, on the mechanical properties of MPC (Table S2).

Table S2. Effect of CS polymer incorporation without a gelation agent on mechanical properties of MPC ($n = 7$, data are expressed as the mean ± SD; No statistically significant difference compared with MPC, wherever the corresponding symbol is not displayed.

Cement	Compressive strength (MPa)	Young Modulus (GPa)
MPC	20.92 ± 6.58	1.86 ± 0.60
MPC+1.5%CS	26.48 ± 4.59	1.84 ± 0.29
